# Comparison of fatigue, cognitive dysfunction and psychological disorders in post-COVID patients and patients after sepsis: is there a specific constellation?

**DOI:** 10.1007/s15010-021-01733-3

**Published:** 2022-01-07

**Authors:** Andreas Stallmach, Miriam Kesselmeier, Michael Bauer, Judith Gramlich, Kathrin Finke, Anne Fischer, Carolin Fleischmann-Struzek, Astrid Heutelbeck, Katrin Katzer, Stephanie Mutschke, Mathias W. Pletz, Stefanie Quickert, Konrad Reinhart, Zoe Stallmach, Martin Walter, André Scherag, Philipp A. Reuken

**Affiliations:** 1 Department of Internal Medicine IV (Gastroenterology, Hepatology and Infectious Diseases), Jena University Hospital/Friedrich-Schiller-University Jena, Am Klinikum 1, 07743 Jena, Germany; 2 Center for Sepsis Control and Care (CSCC), Jena University Hospital/Friedrich-Schiller-University Jena, Jena, Germany; 3 Institute of Medical Statistics, Computer and Data Sciences, Jena University Hospital/Friedrich-Schiller-University Jena, Jena, Germany; 4 Department of Anesthesiology and Intensive Care Medicine, Jena University Hospital/Friedrich-Schiller-University Jena, Jena, Germany; 5 Department of Neurology, Jena University Hospital/Friedrich-Schiller-University Jena, Jena, Germany; 6 Institute for Infectious Diseases and Infection Control, Jena University Hospital/Friedrich-Schiller-University Jena, Jena, Germany; 7 Occupational, Social and Environmental Medicine, Jena University Hospital/Friedrich-Schiller-University Jena, Jena, Germany; 8 Department of Anesthesiology and Intensive Care Medicine, Charité-Universitätsmedizin Berlin, Berlin, Germany; 9 Department of Psychiatry, Jena University Hospital/Friedrich-Schiller-University Jena, Jena, Germany

**Keywords:** Post-COVID, Long-COVID, COVID-19, SARS-CoV2, Sepsis

## Abstract

**Background:**

Sequelae of COVID-19 can be severe and longlasting. We compared frequencies of fatigue, depression and cognitive dysfunction in survivors of SARS-CoV-2-infection and sepsis.

**Methods:**

We performed a prospective cohort study of 355 symptomatic post-COVID patients who visited our out-patient clinic for post-COVID-19 care. We compared them with 272 symptomatic patients from the Mid-German Sepsis Cohort, which investigates the long-term courses of sepsis survivors. Possible predictors for frequent clinical findings (fatigue, signs of depression, cognitive dysfunction) in post-COVID were investigated with multivariable logistic regression.

**Results:**

Median age of the post-COVID patients was 51 years (range 17–86), 60.0% were female, and 31.8% required hospitalization during acute COVID-19. In the post-COVID patients (median follow-up time: 163 days) and the post-sepsis patients (180 days), fatigue was found in 93.2% and 67.8%, signs of depression were found in 81.3% and 10.9%, and cognitive dysfunction was found in 23.5% and 21.3%, respectively. In post-COVID, we did not observe an association between fatigue or depression and the severity of acute COVID-19. In contrast, cognitive dysfunction was associated with hospitalization (out-patient versus in-patient) and more frequent in post-COVID patients treated on an ICU compared to the MSC patients.

**Conclusion:**

In post-COVID patients, fatigue and signs of depression are more common than in sepsis survivors, independent from the acute SARS-CoV-2-infection. In contrast, cognitive dysfunction is associated with hospitalization. Despite the differences in frequencies, owing to the similarity of post-COVID and post-sepsis sequelae, this knowledge may help in implementing follow-up approaches after SARS-CoV-2 infection.

**Supplementary Information:**

The online version contains supplementary material available at 10.1007/s15010-021-01733-3.

## Introduction

In December 2019, a new form of severe acute respiratory syndrome (SARS) caused by a new coronavirus (SARS-CoV-2) was first described in Wuhan, China. In March 2020, after spreading worldwide, SARS-CoV-2 was announced as a global pandemic by the World Health Organization (WHO) [1, 2]. Since the beginning of the SARS-CoV-2 pandemic, more than 242.7million people have been infected, and 4.9 million have died (https://covid19.who.int, accessed on October 25th 2021). Although the infection was initially considered to cause a respiratory disease, infections caused by SARS-CoV-2 were subsequently found to elicit a broad spectrum of symptoms. Most patients have either no symptoms or symptoms comparable to those of a mild cold [3]; in approximately 10–15% of patients, severe symptoms including multi-organ dysfunction develop [4].

However, some patients develop long-term sequelae, which can last several months or more. New symptoms can also occur in that period. If their symptoms last more than 12 weeks, patients are considered to suffer from “post-COVID-syndrome” [5], a multisystemic disease including respiratory, psychiatric, cognitive, cardiovascular, gastrointestinal and inflammatory symptoms differing in intensity, frequency and duration. Overall, more than 50 symptoms have been described, most frequently including chronic fatigue, headache, cognitive dysfunction and dyspnea [6]. These findings are supported by a recent international cohort study of 3762 patients, which has reported fatigue and cognitive dysfunction as the most frequent symptoms in patients with post-COVID-syndrome [7]. Additionally, in the current literature, besides the broad range of symptoms, varying frequencies of post-COVID-syndrome have been reported. A recent meta-analysis has reported that 80% (95% confidence interval 65–92%) of all patients develop long-term-sequelae [6]. In contrast, a British cohort study of 4,182 patients with previous SARS-CoV-2 infection has reported that only 13.3% of patients have symptoms lasting longer than 28 days [8]. In a recent German cohort including 958 patients with initial mild course, any symptoms was found in 27.8% and fatigue in 9.7% of the patients 4 months after infection [9].

Long-term sequelae after infections—including bacterial and viral infections, particularly sepsis—have long been known and have been described as a “hidden public health disaster” [10] including fatigue in up to 50% of critically ill patients [11]. These sequelae reduce the reported quality of life [12]. Whether quantitative and qualitative differences exist between patients with long-term sequelae after SARS-CoV-2 infection and those with sequelae after sepsis is currently unknown. Therefore, the aim of our study was to analyze a single-center cohort of post-COVID patients and to compare them to patients included in the Mid-German Sepsis Cohort (MSC) [13] a cohort of patients with sepsis or septic shock treated in an intensive care unit (ICU), particularly with respect to the frequency of three common symptoms (fatigue, depression, cognitive dysfunction). Furthermore, we sought to identify predictors of these three symptoms in patients with post-COVID-syndrome.

## Methods

We prospectively included all patients who presented until July 22nd 2021 in the post-COVID out-patient clinic of the Jena University Hospital (Jena, Germany; established in August 2020). Patients received a structured examination consisting of evaluation of current and initial symptoms, treatment of the SARS-CoV-2 infection, body examination, amnestic information including pre-existing health conditions and social environment and structured psychiatric and cognitive screening. The infection severity was classified with a modified 8-point scale from the WHO [14]. Details on the psychiatric and cognitive screening are provided below.

To compare patients with post-COVID-syndrome with patients overcoming a different severe systemic infectious disease, we used data from patients after sepsis or septic shock who participated in the MSC. Those patients underwent a comparable psychiatric and cognitive screening procedure (see below). Furthermore, potential sepsis sequelae in patient-reported terms (e.g., ‘tingling’ for paresthesia, ‘weakness of memory’ or ‘difficulty to concentrate’ for cognitive impairments) were assessed. Details on the recruitment and phenotypes of these patients can be found in the published cohort profile [13]. To minimize a selection bias in the comparison of symptomatic post-COVID patients with sepsis survivors, we included the subgroup (*n* = 272) of former sepsis patients of the Jena University Hospital (*n* = 290), who reported at least one potential sepsis sequelae in patient-reported terms. These included common post-sepsis impairments, such as cognitive impairments, CIP/CIM, fatigue, psychological impairments, dysphagia or pain.

The institutional ethics committee of Friedrich-Schiller-University Jena approved both studies (2020-1978-Daten, 4669-01/16).

### Neuropsychiatric evaluation—assessments

Patients with post-COVID-syndrome received a structured assessment of fatigue (Fatigue Assessment Scale, FAS; Brief Fatigue Inventory, BFI) [15, 16], depression (Depression module of the Patient Health Questionnaire, PHQ-9) [17] and cognitive dysfunction (“Montreal Cognitive Assessment” (MoCA) screening) [18]. All questionnaires and tests were used in the German version and interpretation was performed according to the manuals. In case a patient answered more than one of the fatigue questionnaires, the worst result was used.

In the MSC, participants also received a structured assessment of fatigue (Chalder Fatigue Scale, CFS) [19] depression (short version of the Brief Symptom Inventory, BSI-18) [20, 21] and cognitive dysfunction (short version of the MoCA via telephone, tMoCA). In the tMoCA, the items requiring drawing or visual impressions were skipped. Maximum number of points was reduced from 30 to 22 [22].

### Neuropsychiatric evaluation—diagnostic findings

For patients with post-COVID-syndrome, a fatigue was assumed when patients had ≥ 22 points in the FAS or a mean level of ≥ 1 in the BFI. Severity of fatigue was defined via the mean BFI (< 1: no Fatigue, 1 to < 4: mild Fatigue, 4 to < 7: moderate Fatigue, 7–10: severe Fatigue) [15, 23, 24]. Depression was defined as minimal or absent when patients had ≤ 4 points in the PHQ-9, as mild when the patients reached 5–9 points, as moderate in the range of 10–14 points and as severe if the patients had ≥ 15 points [25]. Results of ≥ 5 points in the PHQ-9 were regarded as clinically relevant. Cognitive dysfunction was defined for a MoCA test results < 26 points [18].

For MSC patients, a fatigue was assessed based on the dichotomized score of the CFS (value 0 in case of the response “less than usual” or “”no more than usual”, value 1 in case of “more than usual” or “much more than usual”). Then, fatigue was assumed if the patient had ≥ 4 symptoms (points) [26]. For depression, we relied on the BSI-18 subscale for depression and used the age-specific *t* score values provided in [27]. As the norm values [27] were calculated for individuals aged between 60 and 95 years based on a representative sample of the general population of Germany, MSC participants younger than 60 years were mapped to the age group 60–64 years. This applied to 75 participants with a depression subscale evaluation. Then, a depression was assumed if the patient had a *t* score ≥ 63 [28]. Cognitive dysfunction was defined when a patient had ≤ 17 points in the tMoCA [22]. For each instrument, only patients with complete information on the respective score were included.

### Statistical analyses

We summarized the patient characteristics as absolute and relative frequencies for categorical variables, and as medians and first and third quartiles (Q1, Q3) for numerical variables, unless stated otherwise. Relative frequencies were based on patients with information on the respective variable and accompanied by 95% Clopper–Pearson confidence intervals (CI) where necessary. For explorative comparisons between two patient groups, we applied Fisher’s exact test for categorical variables and the Mann–Whitney *U* test for numerical variables. In time to event comparisons, we applied the log rank test and provided the corresponding Kaplan–Meier curve for illustration, if indicated. To investigate associations between frequent diagnostic findings in patients with post-COVID-syndrome (fatigue, depression, and cognitive dysfunction) and the initial severity of the SARS-CoV-2-infection (hospitalization/hospital admission, kind of hospital stay (out-patient only, in-patient on normal ward only, in-patient with ICU admission) and WHO grade), we applied uni- and multivariable logistic regression modelling. For each combination of diagnostic finding and severity, we adjusted for sex and age in a multivariable model. We report (adjusted) odds ratios (ORs) with 95% CIs. We present nominal two-sided *p* values. For the analyses, we used SPSS 26 (IBM Inc, Armonk, NY) and R (version 4.0.2).

## Results

### Overall

Between August 1st 2020 and July 22nd 2021, a total of 355 patients visited our post-COVID out-patient clinic. The median time between infection and visiting the out-patient clinic was 163 (Q1–Q3125–203, range 24–552) days after diagnosis of SARS-CoV-2 infection. Of these patients, 213 were women (60.0%), and the median age was 51 (Q1–Q3: 40–60, range 17–86) years. One-hundred twelve patients (31.8%) required hospitalization for treatment of the SARS-CoV-2 infection. Among the hospitalized patients, 87 (79.8%) required oxygen supply, and 48 (44.4%) were treated in an ICU. According to the WHO ordinal scale, only 11 patients had an initially asymptomatic infection (3.4%, Stage 1). Two-hundred six patients (64.1%) had a mild disease, which could have been treated as out-patients (WHO Stage 2). Twenty-five patients (7.8%) were hospitalized but did not require oxygen supply (WHO Stage 3). Another 37 patients (11.5%) required low-flow oxygen supply because of the SARS-CoV-2 infection (WHO Stage 4). Twenty-four patients (7.5%) required high flow oxygen or non-invasive ventilation (WHO Stage 5). Seventeen patients (5.3%) required organ support, including eight patients (2.5%) with invasive mechanical ventilation (WHO Stage 6) and nine patients (2.8%) with additional support for other organs (ECMO and/or dialysis; WHO Stage 7). Detailed information on demographics and the course of the initial infection is presented in Table 1. An overview of frequently self-reported symptoms is given in Supplemental Table 1.

**Table 1:** Demographics and disease course of post-COVID patients

Characteristic	Number of patients with missing information	Distribution (*N* = 355)
Female sex; *n* (%)	0	213 (60.0%)
Age, in years; median (Q1, Q3), Minimum–Maximum	0	51 (40, 60), 17–86
Days since infection; median (Q1, Q3)	7	163 (125, 203)
Out-patient only; *n* (%)	3	240 (68.2%)
Among them		
WHO grade	23	
1; *n* (%)		11 (5.1%)
2; *n* (%)		206 (94.9%)
In-patient; *n* (%)	3	112 (31.8%)
Among them		
In need of oxygen support; *n* (%)	3	87 (79.8%)
ICU stay; *n* (%)	4	48 (44.4%)
WHO grade	8	
2^*^; *n* (%)		1 (1.0%)
3; *n* (%)		25 (24.0%)
4; *n* (%)		37 (35.6%)
5; *n* (%)		24 (23.1%)
6; *n* (%)		8 (7.7%)
7; *n* (%)		9 (8.7%)

Absolute (*n*) and relative frequencies (%) or median together with first and third quartile (Q1, Q3) are provided. Relative frequencies are related to patients who provided information on the specific characteristic

*ICU* intensive care unit, *N* number of patients in total, *WHO* World Health Organization

^*^This patient was an in-patient for other reasons than SARS-CoV-2 infection

### Neuro-psychiatric and cognitive screening

**Fig. 1:**
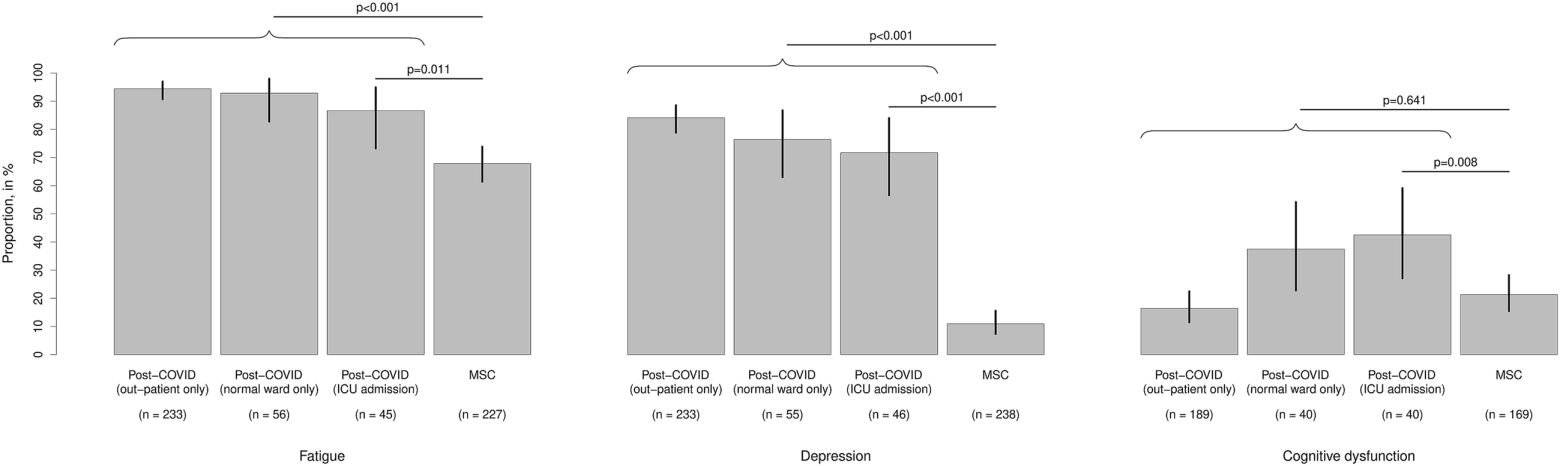
Relative frequencies (grey bar) of psychiatric and cognitive diagnostic findings of patients suffering from the post-COVID-syndrome (stratified by kind of hospital stay) and patients after intensive cared sepsis or septic shock (MSC, Mid-German Sepsis Cohort). Additionally, 95% confidence intervals (black, vertical segments) are given. Percentage refers to patients who provided information for the respective finding (number provided below the bars). In addition, *p* values from comparing post-COVID patients with MSC patients are provided above the bars. All post-COVID patients as well as the subgroup of post-COVID patients with ICU stay are compared with post-sepsis patients (all with ICU stay). Of note, all patients with information on the respective finding are included in the comparison of all post-COVID patients with the MSC patients, although four post-COVID patients with fatigue, three with depression and three with cognitive dysfunction could not be included in the bars due to missing information on their hospital stay. Data are related to the time point 5–6 months after the disease. *ICU* intensive care unit

The structured psychiatric screening revealed pathological results as fatigue or signs of depression in 320 patients (90.1% of all patients). Chronic fatigue was found in 315 of 338 patients (93.2%), including 111 patients (35.2%) with “mild fatigue”, 150 patients (47.6%) with “moderate fatigue” and 53 patients (16.8%) with “severe fatigue”. Signs of depression were found in 274 of 337 patients (81.3%), including 99 patients (36.1%) with “mild” symptoms, 123 patients (44.9%) with “moderate” symptoms and 52 patients (19.0%) with “severe” symptoms. We found a large overlap between fatigue and depression: only 43 patients with fatigue had no signs of depression, and only 4 patients had signs of depression without signs of fatigue. The cognitive screening with the MoCA score indicated cognitive dysfunction in 64 of 272 patients tested (23.5%; Fig. 1).

Then, we analyzed possible predictors (in terms of the initial severity of the SARS-CoV-2 infection) of the frequent diagnostic findings in our post-COVID patients. In univariable analyses (Table 2), we did not observe evidence for an association with possible predictors for fatigue. Signs of depression were more frequent in female patients (OR 0.51, 95% CI 0.29–0.89). Cognitive dysfunction (as indicated by the MoCA score) was associated with age (OR per year 1.05, 95% CI 1.03–1.07), need for hospitalization (OR 3.36, 95% CI 1.88–6.06), kind of hospital stay (in-patient (normal ward) versus out-patient: OR 3.06, 95% CI 1.43–6.43; in-patient (ICU admission) versus out-patient: OR 3.77, 95% CI 1.79–7.87) and the severity as defined by WHO grade (OR per grade increase 1.35, 95% CI 1.11–1.66). After adjusting for age and sex, cognitive dysfunction was still associated with hospitalization (adjusted OR 2.13, 95% CI 1.06–4.27; i.e., more frequent in case of a hospital admission) and age (adjusted OR per year 1.04, 95% CI 1.01–1.06). We did not observe evidence of an association of the possible predictors with depression or fatigue (Table 2). In Fig. 1, we provide the diagnostic findings, stratified by kind of hospital stay (out-patient treatment, hospitalization without ICU treatment and ICU treatment). In contrast, the length of hospital stay among post-COVID patients (including about 68% out-patients and 32% in-patients) was longer in patients with cognitive dysfunction [median of 3 (Q1–Q3 0–15) days compared with 0 (Q1–Q3 0–0) days] and associated with depression [median of 0 (Q1–Q3 0–5) days compared with median of 0 (Q1–Q3 0–12) days]. For depression, this also holds true when considering only in-patients. We could not observe evidence for an association of fatigue with length of hospital stay. The corresponding overview is provided in Supplemental Table 2 as well as in Supplemental Fig. 1.

**Table 2:** Results of uni- and multivariable logistic regression modelling to assess associations between frequent diagnostic findings (fatigue, depression, cognitive dysfunction) in patients suffering from post-COVID-syndrome and the initial severity of the COVID disease (hospital admission, kind of hospital stay, WHO grade)

Variable	Univariable		Multivariable (adjusted for age and sex)	
	OR (95% CI)	*p* value	Adjusted OR (95% CI)	*p* value
Finding: fatigue				
Hospital admission (ref.: no)	0.56 (0.24, 1.34)	0.180	0.67 (0.25, 1.87)	0.439
Kind of hospital stay (ref.: out-patient only)		0.223		0.491
In-patient (normal ward only)	0.77 (0.26, 2.81)	0.656	0.84 (0.27, 3.23)	0.778
In patient (ICU admission)	0.38 (0.14, 1.15)	0.067	0.46 (0.13, 1.69)	0.229
WHO grade, per grade	0.78 (0.59, 1.05)	0.081	0.86 (0.61, 1.24)	0.406
Sex (ref.: female)	0.68 (0.29, 1.61)	0.374	–	–
Age, in years	0.98 (0.96, 1.01)	0.278	–	–
Finding: depression				
Hospital admission (ref.: no)	0.57 (0.32, 1.00)	0.049	0.72 (0.37, 1.40)	0.326
Kind of hospital stay (ref.: out-patient only)		0.102		0.538
In-patient (normal ward only)	0.61 (0.30, 1.28)	0.175	0.70 (0.33, 1.53)	0.355
In patient (ICU admission)	0.48 (0.23, 1.02)	0.049	0.67 (0.29, 1.61)	0.361
WHO grade, per grade	0.85 (0.70, 1.04)	0.109	0.95 (0.75, 1.21)	0.669
Sex (ref.: female)	0.51 (0.29, 0.89)	0.018	–	–
Age, in years	0.99 (0.97, 1.01)	0.197	–	–
Finding: cognitive dysfunction				
Hospital admission (ref.: no)	3.36 (1.88, 6.06)	< 0.001	2.13 (1.06, 4.27)	0.033
Kind of hospital stay (ref.: out-patient only)		< 0.001		0.109
In-patient (normal ward only)	3.06 (1.43, 6.43)	0.003	2.04 (0.89, 4.53)	0.085
In patient (ICU admission)	3.77 (1.79, 7.87)	< 0.001	2.23 (0.91, 5.46)	0.077
WHO grade, per grade	1.35 (1.11, 1.66)	0.003	1.14 (0.89, 1.46)	0.307
Sex (ref.: female)	1.14 (0.64, 2.01)	0.653	–	–
Age, in years	1.05 (1.03, 1.07)	< 0.001	–	–

(Adjusted) odds ratio (OR) with 95% confidence interval (CI) and *p* value are given. For categorical variables, the reference category (ref.) is provided. The complete results are provided in Supplemental Table 3

*ICU* intensive care unit, *WHO* World Health Organization

Because post-COVID patients mostly arrived at the out-patient clinic if they experienced symptoms, we compared our patients to the 272 patients with at least one reported potential sepsis sequelae (in patient-reported terms) out of the 290 patients from Jena included in the MSC (104 females (38.2%), median age of 66 (Q1–Q3 57–75; range 19–93) years). During their hospital stay, 231 patients (85.0%) had a septic shock and 215 patients (79.0%) required at least one organ replacement therapy (204 patients (75.0%) mechanical ventilation including noninvasive ventilation, one patient (0.4%) ECMO, 71 patients (26.2%) dialysis, one patient (0.4%) other organ replacement therapy). All these patients were treated in an ICU because of sepsis, an inclusion criterion for the MSC, and were followed up for 6 months after ICU discharge. At the follow-up assessment, fatigue was found in 154 of 227 patients (67.8%), signs of depression were found in 26 out of 238 patients (10.9%), and pathological results in the tMoCA score were found in 36 of 169 patients (21.3%). Comparing the MSC patients with post-COVID patients admitted to an ICU, fatigue, signs of depression and cognitive dysfunction were more frequent in post-COVID patients (Fig. 1). When defining the length of hospital stay as a parameter for the severity of illness in the MSC patients, depression was associated with a longer length of hospital stay (median of 40 (Q1–Q3 28–65) days compared with median of 32 (Q1–Q3 21–46) days), but we did not observe an association between fatigue or cognitive dysfunction and the length of hospital stay (Supplemental Table 2, Supplemental Fig. 1).

## Discussion

The aim of our study in post-COVID patients who visited our out-patient clinic for post-COVID-19 care was twofold: (i) comparison with symptomatic patients after sepsis or septic shock as an example of another severe infection and (ii) identification of predictors of three frequent clinical findings in post-COVID patients, namely fatigue, depression and cognitive dysfunction.

Comparing frequencies of long-term sequelae between symptomatic post-COVID and post-sepsis patients, fatigue and signs of depression were more frequent in post-COVID patients (overall and ICU-treated). For cognitive dysfunction, we could not observe evidence for overall differences between post-COVID and post-sepsis patients, but it was more frequent in the subgroup of post-COVID patients treated on an ICU than in MSC participants. In post-COVID patients, cognitive dysfunction was associated with hospitalization and age. However, a recently published study reported cognitive dysfunction in 14 out of 18 patients with an initial mild course of COVID-19. These patients visited an out-patients clinic similar to our patients [29]. In this context, it is important to note, that this patient group is rather small. In a large cohort of 236,379 patients following a SARS-CoV-2 infection, neurological or psychiatric sequelae based on ICD-10 codes up to day 180 were reported in 12.8% of the patients excluding patients already suffering from that disease pre-infection, and showed—similar to our data—a trend to be more frequently in more severe ill patients [30].

In our cohort, 11 patients developed symptoms after an initial asymptomatic course of COVID-19. The extent to which post-COVID-syndrome occurs in these patients remains unclear, because the current literature has examined either selected cohorts, e.g., hospitalized or ICU-treated patients, or patients after symptom-driven medical contact, as in our study. Gold et al. found 4 out of 56 symptomatic patients having an initial asymptomatic infection (7.1%) [31]. As a result, patients without post-COVID symptoms are under-represented in both settings, thus potentially leading to an artificially high rate of symptomatic patients. Population-based cohorts using health authority or insurance data on positive tests are urgently needed to estimate the burden of post-COVID-syndrome (both incidence of the syndrome and of the symptoms). Post-viral fatigue is not only restricted to post-COVID patients, but also common after other viral infections. Following an infection with SARS-CoV-1 or MERS-CoV, up to 15% of the patients develop neuropsychiatric symptoms, which was less frequent than in patients after SARS-CoV-2 infection.

In most patients suffering from sequelae associated with SARS-CoV-1 or MERS-CoV infection, these symptoms resolved without residual problems [32]; contrasting, given the limited evidence due to the nature of a “new disease”, for several cohorts symptoms following a SARS-CoV-2 infection lasting for 7 to 12 month were reported [7, 33]. In line with these reports, we did not observe significant differences in patients depending on the time since infection (data not shown).

In a historic cohort of 31 patients presenting with chronic fatigue, Strauss et al. found evidence for persisting EBV infection in 23 of the patients (74.2%) [34]. Persistence of the SARS-CoV-2 virus with ongoing immune response as a pathophysiologic mechanism is also discussed in SARS-CoV-2 patients with long-lasting symptoms. Files et al. reported increased immune response against SARS-CoV-2 with respect to both antibodies and specific T cells [35]. Of note, two of our patients (0.6%, data not shown) had repeated positive SARS-CoV-2 polymerase chain reaction (PCR) results at presentation in the out-patient clinic and both of them reported persistent fatigue. However, in clinical routine, we only performed SARS-CoV-2 PCR tests in nose and mouth, but viral persistence outside the respiratory system may also be important in post-COVID patients, e.g., endothelium of small blood vessels, but cannot be ruled out with our approach.

The main strength of our study might also be regarded as the main limitation, as we mainly included patients with symptoms (due to relying on patients presenting at our out-patient clinic). Within this group of patients, it is possible to assess the distribution of symptoms but not the incidence of (any) post-COVID-syndrome symptoms. This is also an issue in the comparison of post-COVID with post-sepsis patients, because all surviving post-sepsis patients were included in the MSC. Hence, we selected the subgroup of MSC patients who reported potential sepsis sequelae (in patient reported terms). Furthermore, slight differences exist in the design of the evaluation instruments and the different recruitment periods (the MSC recruitment occurred before the pandemic). In some parts, another limitation is that the majority of post-COVID patients were treated as out-patients only, which might hinder the identification of associations of diagnostic findings with disease severity, probably aggravated for fatigue by the high proportion of patients suffering from fatigue.

In conclusion, post-COVID symptoms affect a large proportion of COVID-19 survivors, pattern of symptoms is similar to sequelae in sepsis survivors, but these are more common in post-COVID patients. Because of the nature of a “new” disease, it is unknown whether these symptoms can also last for longer time periods, such as years. Given the large number of SARS-CoV-2 infections during the pandemic, post-COVID-syndrome will become a major public health issue and concepts to address problems of these patients are urgently needed. Nevertheless, despite the differences in frequencies, owing to the similarity of the spectrum of post-COVID and post-sepsis sequelae, this knowledge may help physicians in implementing follow-up and therapeutic approaches for patients after SARS-CoV-2 infection [12]. Up today, despite the growing evidence for large proportion of patients suffering from long-term sequelae, there is no structured follow-up for these patients, but should be implemented, e.g., using questionnaires reporting neuropsychiatric or somatic symptoms.

## Supplementary Information

Below is the link to the electronic supplementary material.

Supplementary file1 (DOCX 311 KB)

## References

[1] Zhu N, Zhang D, Wang W, Li X, Yang B, Song J et al. A novel Coronavirus from patients with pneumonia in China, 2019. N Engl J Med [Internet]. 2020: https://www.nejm.org/doi/10.1056/NEJMoa2001017. Cited 25 Apr 2020.

[2] WHO Director-General’s opening remarks at the media briefing on COVID-19—11 March 2020 [Internet]. https://www.who.int/dg/speeches/detail/who-director-general-s-opening-remarks-at-the-media-briefing-on-covid-19---11-march-2020. Cited 25 Apr 2020.

[3] Wu Z, McGoogan JM (2020). Characteristics of and important lessons from the coronavirus disease 2019 (COVID-19) outbreak in China: summary of a report of 72 314 cases from the Chinese Center for Disease Control and Prevention. JAMA.

[4] Zhou F, Yu T, Du R, Fan G, Liu Y, Liu Z (2020). Clinical course and risk factors for mortality of adult inpatients with COVID-19 in Wuhan, China: a retrospective cohort study. Lancet Lond Engl..

[5] NICE Guideline, No. 188. COVID-19 rapid guideline: managing the long-term effects of COVID-19 [Internet]. London: National Institute for Health and Care Excellence (UK); 2020 (National Institute for Health and Care Excellence: Clinical Guidelines). Available from: http://www.ncbi.nlm.nih.gov/books/NBK567261/. Cited 5 May 2021.

[6] Lopez-Leon S, Wegman-Ostrosky T, Perelman C, Sepulveda R, Rebolledo P, Cuapio A et al. More than 50 long-term effects of COVID-19: a systematic review and meta-analysis [Internet]. In Review; 2021: https://www.researchsquare.com/article/rs-266574/v1. Cited 24 Mar 2021. 10.1038/s41598-021-95565-8 PMC8352980 34373540

[7] Davis HE, Assaf GS, McCorkell L, Wei H, Low RJ, Re’em Y et al. Characterizing long COVID in an international cohort: 7 months of symptoms and their impact [Internet]. Infect Dis (except HIV/AIDS); 2020. http://medrxiv.org/lookup/doi/10.1101/2020.12.24.20248802. Cited 24 Mar 2021. 10.1016/j.eclinm.2021.101019 PMC8280690 34308300

[8] Sudre CH, Murray B, Varsavsky T, Graham MS, Penfold RS, Bowyer RC et al. Attributes and predictors of long COVID. Nat Med [Internet]. 2021: http://www.nature.com/articles/s41591-021-01292-y. Cited 24 Mar 2021. 10.1038/s41591-021-01292-y PMC7611399 33692530

[9] Augustin M, Schommers P, Stecher M, Dewald F, Gieselmann L, Gruell H (2021). Post-COVID syndrome in non-hospitalised patients with COVID-19: a longitudinal prospective cohort study. Lancet Reg Health Eur..

[10] Angus DC (2010). The lingering consequences of sepsis: a hidden public health disaster?. JAMA.

[11] Wintermann G-B, Rosendahl J, Weidner K, Strauß B, Hinz A, Petrowski K (2018). Self-reported fatigue following intensive care of chronically critically ill patients: a prospective cohort study. J Intensive Care.

[12] König C, Matt B, Kortgen A, Turnbull AE, Hartog CS (2019). What matters most to sepsis survivors: a qualitative analysis to identify specific health-related quality of life domains. Qual Life Res Int J Qual Life Asp Treat Care Rehabil.

[13] Fleischmann-Struzek C, Kesselmeier M, Ouart D, Hartog CS, Bauer M, Bercker S (2021). Mid-German Sepsis Cohort (MSC): a prospective observational study of sepsis survivorship. BMJ Open.

[14] WHO Working Group on the Clinical Characterisation and Management of COVID-19 Infection (2020). A minimal common outcome measure set for COVID-19 clinical research. Lancet Infect Dis.

[15] Mendoza TR, Wang XS, Cleeland CS, Morrissey M, Johnson BA, Wendt JK (1999). The rapid assessment of fatigue severity in cancer patients: use of the Brief Fatigue Inventory. Cancer.

[16] Vries J, Michielsen H, Heck GL, Drent M (2004). Measuring fatigue in sarcoidosis: the Fatigue Assessment Scale (FAS). Br J Health Psychol.

[17] Kroenke K, Spitzer RL, Williams JB (2001). The PHQ-9: validity of a brief depression severity measure. J Gen Intern Med.

[18] Nasreddine ZS, Phillips NA, Badirian V, Charbonneau S, Whitehead V, Collin I (2005). The Montreal Cognitive Assessment, MoCA: a brief screening tool for mild cognitive impairment: MOCA: a brief screening tool for MCI. J Am Geriatr Soc.

[19] Chalder T, Berelowitz G, Pawlikowska T, Watts L, Wessely S, Wright D (1993). Development of a fatigue scale. J Psychosom Res.

[20] Derogatis LR (2000). Brief Symptom Inventory (BSI) 18. Administration, scoring and procedures manual.

[21] Franke GH, Jaeger S, Glaesmer H, Barkmann C, Petrowski K, Braehler E (2017). Psychometric analysis of the brief symptom inventory 18 (BSI-18) in a representative German sample. BMC Med Res Methodol.

[22] Katz MJ, Wang C, Nester CO, Derby CA, Zimmerman ME, Lipton RB et al. T‐MoCA: a valid phone screen for cognitive impairment in diverse community samples. Alzheimer’s Dement Diagn Assess Dis Monit [Internet]. 2021;13(1). Available from: https://onlinelibrary.wiley.com/doi/10.1002/dad2.12144. Cited 27 Mar 2021. 10.1002/dad2.12144 PMC7864219 33598528

[23] Radbruch L, Sabatowski R, Elsner F, Everts J, Mendoza T, Cleeland C (2003). Validation of the German version of the brief fatigue inventory. J Pain Symptom Manag.

[24] Yun YH, Wang XS, Lee JS, Roh JW, Lee CG, Lee WS (2005). Validation study of the Korean version of the brief fatigue inventory. J Pain Symptom Manag.

[25] Löwe B, Spitzer R, Zipfel S, Herzog W. Manual—Komplett und Kurzversion Autorisierte deutsche Version des "Prime MD Patient Health Questionnaire (PHQ)"

[26] Martin A, Staufenbiel T, Gaab J, Rief W, Brähler E (2010). Messung chronischer Erschöpfung—Teststatistische Prüfung der Fatigue Skala (FS). Z Für Klin Psychol Psychother.

[27] Petrowski K, Schmalbach B, Jagla M, Franke GH, Brähler E (2018). Norm values and psychometric properties of the brief symptom inventory-18 regarding individuals between the ages of 60 and 95. BMC Med Res Methodol.

[28] Franke H. BSI. Brief Symptom Inventory—Deutsche Version. Manual. Beltz, Göttingen, 2000.

[29] Woo MS, Malsy J, Pöttgen J, Seddiq Zai S, Ufer F, Hadjilaou A (2020). Frequent neurocognitive deficits after recovery from mild COVID-19. Brain Commun..

[30] Taquet M, Geddes JR, Husain M, Luciano S, Harrison PJ (2021). 6-month neurological and psychiatric outcomes in 236 379 survivors of COVID-19: a retrospective cohort study using electronic health records. Lancet Psychiatry.

[31] Gold JE, Okyay RA, Licht WE, Hurley DJ (2021). Investigation of long COVID prevalence and its relationship to Epstein-Barr virus reactivation. Pathog Basel Switz.

[32] Rogers JP, Chesney E, Oliver D, Pollak TA, McGuire P, Fusar-Poli P (2020). Psychiatric and neuropsychiatric presentations associated with severe coronavirus infections: a systematic review and meta-analysis with comparison to the COVID-19 pandemic. Lancet Psychiatry.

[33] Seeßle J, Waterboer T, Hippchen T, Simon J, Kirchner M, Lim A (2021). Persistent symptoms in adult patients one year after COVID-19: a prospective cohort study. Clin Infect Dis.

[34] Straus SE, Tosato G, Armstrong G, Lawley T, Preble OT, Henle W (1985). Persisting illness and fatigue in adults with evidence of Epstein-Barr virus infection. Ann Intern Med.

[35] Files JK, Sarkar S, Fram TR, Boppana S, Sterrett S, Qin K (2021). Duration of post-COVID-19 symptoms is associated with sustained SARS-CoV-2-specific immune responses. JCI Insight..

