# Supplementary material for: Comparison of fatigue, cognitive dysfunction and psychological disorders in post-COVID patients and patients after sepsis: is there a specific constellation?
Source: Infection. 2022 Jan 7;50(3):661–9. doi: 10.1007/s15010-021-01733-3 (PMC8741139; doi:10.1007/s15010-021-01733-3)
Supplement: Supplementary file 1 — Supplementary file1 (DOCX 311 KB) [file 15010_2021_1733_MOESM1_ESM.docx]

**Comparison of fatigue, cognitive dysfunction and psychological disorders in post-COVID patients and patients after sepsis. Is there a specific constellation?**

**Andreas Stallmach^1^**^,^**^2#^**, **Miriam Kesselmeier^2^**^,^**^3#^**, **Michael Bauer^2^**^,^**^4^**, **Judith Gramlich^1^**, **Kathrin Finke^5^**, **Anne Fischer^1^**, **Carolin Fleischmann-Struzek^2^**^,^**^6^**, **Astrid Heutelbeck^7^**, **Katrin Katzer^1^**, **Stephanie Mutschke^1^**, **Mathias W. Pletz^6^**, **Stefanie Quickert^1^, Konrad Reinhart ^2^**^,^**^8^**, **Zoe Stallmach^1^**, **Martin Walter^9^**, **André Scherag^2^**^,^**^3#^**, **Philipp A. Reuken^1#^**

^#^ both first authors and both last authors contributed equally to this work

^1^Department of Internal Medicine IV (Gastroenterology, Hepatology and Infectious Diseases), Jena University Hospital, Jena, Germany

^2^Center for Sepsis Control & Care (CSCC), Jena University Hospital/Friedrich-Schiller-University Jena, Jena, Germany

^3^Institute of Medical Statistics, Computer and Data Sciences, Jena University Hospital/Friedrich-Schiller-University Jena, Jena, Germany

^4^Department of Anesthesiology and Intensive Care Medicine, Jena University Hospital, Jena, Germany

^5^Department of Neurology, Jena University Hospital, Jena, Germany

^6^Institute for Infectious Diseases and Infection Control, Jena University Hospital, Jena, Germany

^7^Occupational, Social and Environmental Medicine, Jena University Hospital, Friedrich-Schiller-University Jena, Jena, Germany

^8^Department of Anesthesiology and Intensive Care Medicine, Charité–Universitätsmedizin Berlin, Berlin

^9^Departement of Psychiatry, Jena University Hospital, Jena, Germany

**Correspondence**

Prof. Dr. A. Stallmach

Klinik für Innere Medizin IV (Gastroenterologie, Hepatologie, Infektiologie)

Universitätsklinikum Jena

Am Klinikum 1

07743 Jena

Germany

Tel.: 0049 (0)3641-9324401

Email: [andreas.stallmach@med.uni-jena.de](mailto:andreas.stallmach@med.uni-jena.de)

**Supplemental Tables**

Supplemental Table 1. Selected somatic symptoms in post-COVID patients

| Characteristic | Number of  (N = 355) | Proportion, in %  (95% CI) |
| --- | --- | --- |
| *Overall* |  |  |
| Symptoms provided^*6^ |  |  |
| none; n (%) | 12 | 3.5 (1.8, 6.0) |
| at least 1; n (%) | 332 | 96.5 (94.0, 98.2) |
| *Patients with symptoms* |  |  |
| Distribution^*1^ |  |  |
| median (Q1, Q3) |  | 4 (2; 6) |
| 1; n (%) | 31 | 9.9 (6.8, 13.8) |
| 2; n (%) | 55 | 17.6 (13.5, 22.2) |
| 3; n (%) | 51 | 16.3 (12.4, 20.9) |
| 4; n (%) | 50 | 16.0 (12.1, 20.5) |
| 5 or above; n (%) | 114 | 36.4 (31.1, 42.0) |
| Frequency of specific symptoms: |  |  |
| limited physical capacity^*2^; n (%) | 275 | 79.0 (74.4, 83.2) |
| dyspnea^*3^; n (%) | 212 | 61.1 (55.7, 66.3) |
| subjective cognitive dysfunction^*3^; n (%) | 202 | 58.2 (52.8, 63.5) |
| odor / taste disorder^*5^; n (%) | 113 | 32.8 (27.8, 38.0) |
| sleep disorder^*5^; n (%) | 144 | 41.7 (36.5, 47.1) |
| arthralgia^*5^; n (%) | 131 | 38.0 (32.8, 43.3) |
| cephalgia^*4^; n (%) | 122 | 35.3 (30.2, 40.5) |
| cough^*5^; n (%) | 70 | 20.3 (16.2, 24.9) |
| abdominal pain^*5^; n (%) | 60 | 17.4 (13.5, 21.8) |
| hair loss^*7^; n (%) | 43 | 13.7 (10.1, 18.1) |

Multiple answers for symptoms possible. Absolute (n) and relative frequencies (%) are provided. Additionally, proportions are accompanied by 95% confidence intervals (CI). Relative frequencies are related to patients who provided information on the specific characteristic. Abbreviations: N, number of patients in total.

^*1^ Information on symptoms missing for 42 patients, as only patients with complete information in symptoms can be considered.

^*2^ Information missing for seven patients.

^*3^ Information missing for eight patients.

^*4^ Information missing for nine patients.

^*5^ Information missing for ten patients.

^*6^ Information missing for eleven patients. In case of provided symptoms “none”, patients stated for all symptoms to report, that they did not occur. In case of provided symptoms “at least 1”, patients did not need to provide information on all symptoms to report, but had to state for at least one symptom that it occurred.

^*7^ Information missing for 42 patients.

Supplemental Table 2. Association between occurrence of a diagnostic finding and length of hospital stay for patients with post-COVID-syndrome (overall and stratified by hospital admission as well as kind of hospital stay) and for patients with status after sepsis or septic shock (Mid-German Sepsis Cohort; MSC)

| Diagnostic finding | N | Length of hospital stay, in days | | p-value |
| --- | --- | --- | --- | --- |
|  |  | Finding present | Finding not present |  |
| *Finding: fatigue* |  |  |  |  |
| Post-COVID |  |  |  |  |
| all patients | 328 | 0 (0, 5) | 0 (0, 12) | 0.126 |
| in-patients (overall) | 100 | 13 (7, 21) | 15 (8, 25) | 0.700 |
| in patients (normal ward only) | 54 | 8 (5, 13) | 11 (8, 17) | 0.388 |
| in patients (ICU admission) | 45 | 22 (15, 34) | 20 (10, 25) | 0.300 |
| MSC | 227 | 33 (22, 48) | 29 (22, 45) | 0.740 |
| *Finding: depression* |  |  |  |  |
| Post-COVID |  |  |  |  |
| all patients | 328 | 0 (0, 5) | 0 (0, 12) | 0.047 |
| in-patients (overall) | 100 | 13 (7, 21) | 13 (8, 22) | 0.813 |
| in patients (normal ward only) | 53 | 8 (5, 13) | 9 (4, 13) | 0.506 |
| in patients (ICU admission) | 46 | 22 (15, 36) | 19 (13, 22) | 0.277 |
| MSC | 238 | 40 (28, 65) | 32 (21, 46) | 0.030 |
| *Finding: cognitive dysfunction* |  |  |  |  |
| Post-COVID |  |  |  |  |
| all patients | 265 | 3 (0, 15) | 0 (0, 0) | <0.001 |
| in-patients (overall) | 265 | 3 (0, 15) | 0 (0, 0) | <0.001 |
| in patients (normal ward only) | 79 | 15 (9, 21) | 13 (7, 22) | 0.525 |
| in patients (ICU admission) | 39 | 9 (8, 16) | 8 (4, 13) | 0.229 |
| MSC | 169 | 28 (21, 42) | 30 (20, 43) | 0.821 |

Median together with first and third quartile (Q1, Q3) as well as the p-value from the Mann-Whitney-U test are provided. Furthermore, the number of patients (N) with information on the respective question is given. Of note, about 68% of the post-COVID patients were out-patients and all MSC patients were in-patients with need of intensive care. Of note, all patients with information on the respective finding are included in the comparison of all post-COVID patients, although four post-COVID patients with fatigue, three with depression and three with cognitive dysfunction could not be included in the comparisons of the subgroups due to missing information on their hospital stay. Abbreviations: ICU, intensive care unit.

Supplemental Table 3. Complete results of multivariable logistic regression modelling (adjusted for sex and age) to assess associations between frequent diagnostic findings (fatigue, depression, cognitive dysfunction) in patients suffering from post-COVID-syndrome and the initial severity of the COVID disease (hospital admission, kind of hospital stay, WHO grade).

| Variable | Fatigue | | Depression | | Cognitive dysfunction | |
| --- | --- | --- | --- | --- | --- | --- |
|  | adjusted OR  (95% CI) | p-value | adjusted OR  (95% CI) | p-value | adjusted OR  (95% CI) | p-value |
|  |  |  |  |  |  |  |
| *Measure: hospital admission* |  |  |  |  |  |  |
| Hospital admission (ref.: no) | 0.67 (0.25, 1.87) | 0.439 | 0.72 (0.37, 1.40) | 0.326 | 2.13 (1.06, 4.27) | 0.033 |
| Sex (ref.: female) | 0.78 (0.32, 1.92) | 0.576 | 0.58 (0.32, 1.03) | 0.061 | 0.77 (0.40, 1.44) | 0.424 |
| Age, in years | 0.99 (0.96, 1.02) | 0.597 | 1.00 (0.97, 1.02) | 0.627 | 1.04 (1.01, 1.06) | 0.003 |
| *Measure: kind of hospital stay* |  |  |  |  |  |  |
| Kind of hospital stay (ref.: out-patient only) |  | 0.491 |  | 0.538 |  | 0.109 |
| in-patient (normal ward only) | 0.84 (0.27, 3.23) | 0.778 | 0.70 (0.33, 1.53) | 0.355 | 2.04 (0.89, 4.53) | 0.085 |
| in-patient (ICU admission) | 0.46 (0.13, 1.69) | 0.229 | 0.67 (0.29, 1.61) | 0.361 | 2.23 (0.91, 5.46) | 0.077 |
| Sex (ref.: female) | 0.81 (0.33, 2.07) | 0.657 | 0.56 (0.31, 1.02) | 0.056 | 0.76 (0.39, 1.45) | 0.415 |
| Age, in years | 0.99 (0.96, 1.03) | 0.742 | 1.00 (0.98, 1.02) | 0.675 | 1.04 (1.01, 1.06) | 0.003 |
| *Measure: WHO grade* |  |  |  |  |  |  |
| WHO grade, per grade | 0.86 (0.61, 1.24) | 0.406 | 0.95 (0.75, 1.21) | 0.669 | 1.14 (0.89, 1.46) | 0.307 |
| Sex (ref.: female) | 0.79 (0.31, 2.08) | 0.630 | 0.50 (0.27, 0.91) | 0.023 | 0.66 (0.33, 1.29) | 0.235 |
| Age, in years | 0.98 (0.95, 1.02) | 0.372 | 0.99 (0.97, 1.01) | 0.565 | 1.04 (1.02, 1.07) | 0.001 |

Adjusted odds ratio (OR) with 95% confidence interval (CI) and p-value are given. For categorical variables, the reference category (ref.) is provided. Abbreviations: ICU, intensive care unit; WHO, World Health Organization.

**Supplemental Figures**


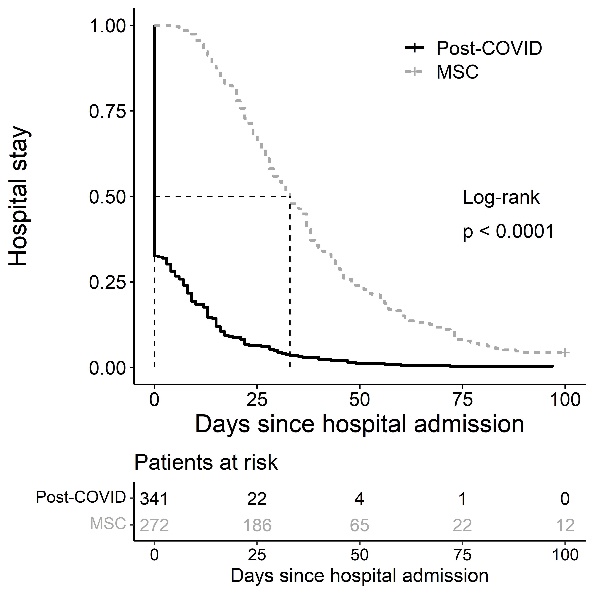

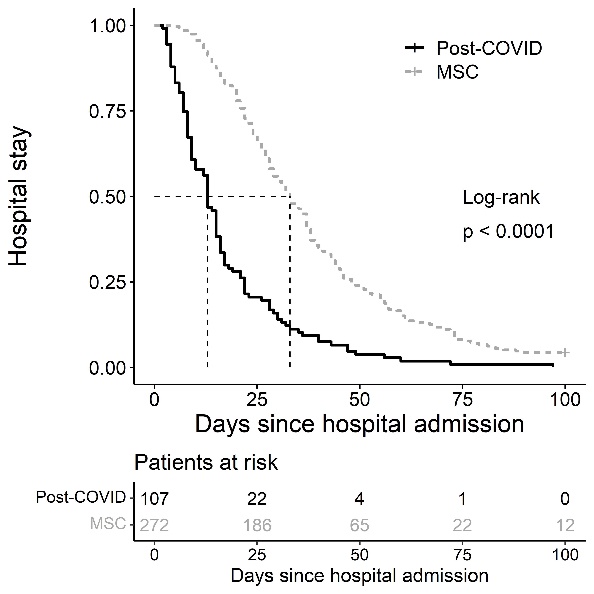


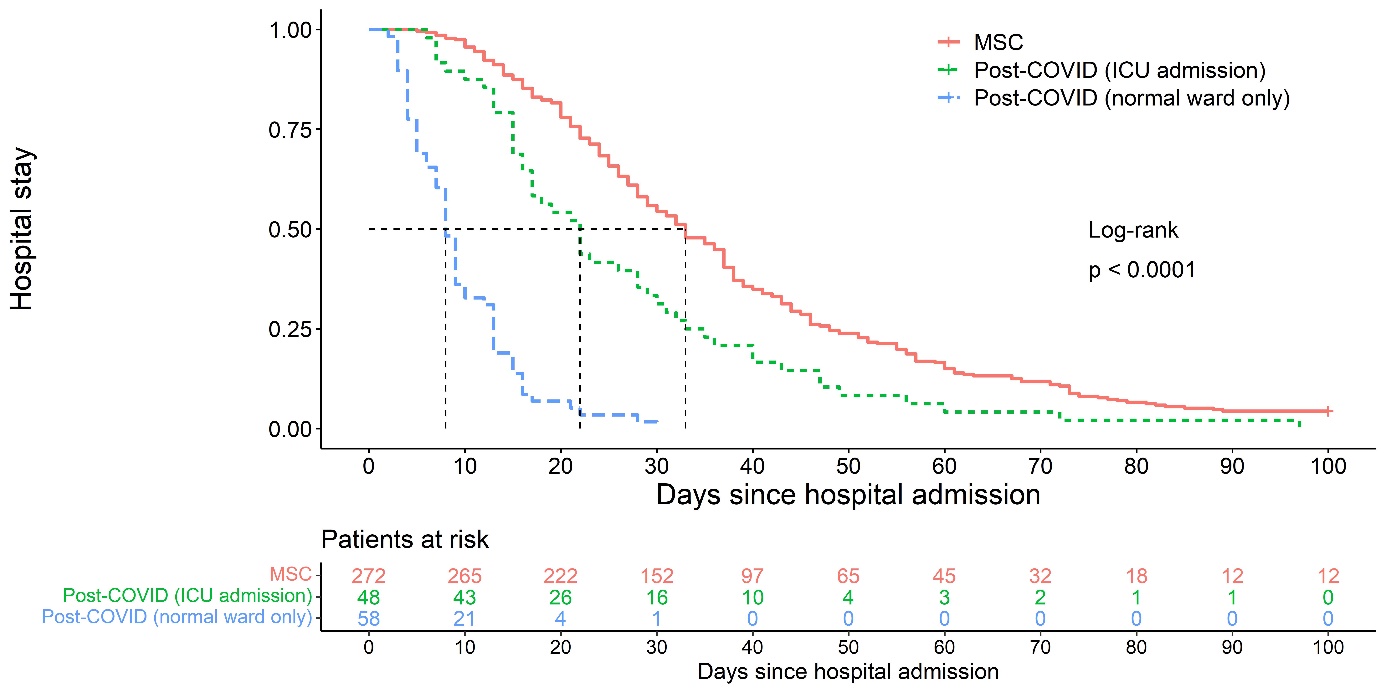


Supplemental Figure 1. Length of hospital stay of patients with post-COVID-syndrome and those with status after sepsis or septic shock (Mid-German Sepsis Cohort, MSC). For post-COVID-syndrome, both all patients (left upper panel) and in-patients only (right upper panel) are provided. Furthermore, information is provided for in-patients stratified by need of intensive care (lower panel). All MSC patients were in-patients and required an intensive care unit (ICU) stay. The length of stay was censored at 100 days. The median length of stay are indicated as dashed line. In addition, the p-value of the log-rank test is provided. The number of patients at risk at selected time points are provided below the Kaplan-Meier curve.
